# Prevention of Pregnancy Complications Using a Multimodal Lifestyle, Screening, and Medical Model

**DOI:** 10.3390/jcm13154344

**Published:** 2024-07-25

**Authors:** Jim Parker, Pierre Hofstee, Shaun Brennecke

**Affiliations:** 1 School of Medicine, University of Wollongong, Wollongong 2522, Australia; pierre.hofstee@health.nsw.gov.au; 2 Tweed Hospital, Northern New South Wales Local Health District, Tweed Heads 2485, Australia; 3 Department of Maternal-Fetal Medicine, Pregnancy Research Centre, The Royal Women’s Hospital, Melbourne 3052, Australia; s.brennecke@unimelb.edu.au; 4 Department of Obstetrics and Gynaecology, The University of Melbourne, Melbourne 3052, Australia

**Keywords:** pre-eclampsia, fetal growth restriction, preterm labor, stillbirth, lifestyle, nutrition, exercise, multivariate screening, angiogenic ratio, aspirin

## Abstract

Prevention of pregnancy complications related to the “great obstetrical syndromes” (preeclampsia, fetal growth restriction, spontaneous preterm labor, and stillbirth) is a global research and clinical management priority. These syndromes share many common pathophysiological mechanisms that may contribute to altered placental development and function. The resulting adverse pregnancy outcomes are associated with increased maternal and perinatal morbidity and mortality and increased post-partum risk of cardiometabolic disease. Maternal nutritional and environmental factors are known to play a significant role in altering bidirectional communication between fetal-derived trophoblast cells and maternal decidual cells and contribute to abnormal placentation. As a result, lifestyle-based interventions have increasingly been recommended before, during, and after pregnancy, in order to reduce maternal and perinatal morbidity and mortality and decrease long-term risk. Antenatal screening strategies have been developed following extensive studies in diverse populations. Multivariate preeclampsia screening using a combination of maternal, biophysical, and serum biochemical markers is recommended at 11–14 weeks’ gestation and can be performed at the same time as the first-trimester ultrasound and blood tests. Women identified as high-risk can be offered prophylactic low dose aspirin and monitored with angiogenic factor assessment from 22 weeks’ gestation, in combination with clinical assessment, serum biochemistry, and ultrasound. Lifestyle factors can be reassessed during counseling related to antenatal screening interventions. The integration of lifestyle interventions, pregnancy screening, and medical management represents a conceptual advance in pregnancy care that has the potential to significantly reduce pregnancy complications and associated later life cardiometabolic adverse outcomes.

## 1. Introduction

The combined impact of obstetric syndromes related to placental dysfunction makes a significant contribution to global maternal and perinatal morbidity and mortality [1,2,3]. As a result, considerable international effort has been directed at understanding the pathogenesis and pathophysiology of these related conditions to inform prevention and management strategies. Given the heterogeneity of causes of placental dysfunction, it is recognized that a clinically effective approach to prevention and treatment will involve a multimodal intervention strategy [4,5]. The introduction of a combined approach using lifestyle, screening, and medical management, as outlined in this review, has the potential to significantly reduce morbidity and mortality, decrease transgenerational transmission of chronic disease, and improve long-term maternal and neonatal health following pregnancy [6].

The causes of placental dysfunction can be genetic, epigenetic, or environmental. In recent times, there has been increasing interest in the contribution of pre-existing maternal pathophysiological processes, such as insulin resistance, low-grade chronic inflammation, and hyperandrogenism, to placental disorders [4,7]. This has resulted in renewed interest in the role of lifestyle interventions for the prevention and treatment of pregnancy complications. Lifestyle-based interventions encompass a variety of domains including nutrition, exercise, smoking cessation, weight management, sleep, stress, and community support. Most of the research has been directed to interventions related to diet and exercise, both of which have been recommended by international obstetric societies to promote healthy pregnancy and reduce adverse outcomes [8,9].

First- and second-trimester screening strategies have been developed following rigorous pre-clinical, case-control, observational, intervention, and real-world evaluation studies [10,11]. These screening models have been found to have good predictive value for early diagnosis of some obstetric syndromes, such as pre-eclampsia (PE) and fetal growth restriction (FGR), and result in increased surveillance of all women identified as high-risk [11,12]. Multivariate screening can be performed at 11–14 weeks’ gestation using a combination of maternal, biophysical, and serum biochemical markers [10]. Women identified as high-risk for PE on first-trimester multivariate screening can be offered prophylactic treatment with low-dose aspirin [13]. High-risk women can be monitored by measurement of the serum angiogenic ratio, which can be used in conjunction with ultrasound scans, blood tests, fetal monitoring, and clinical assessment, commencing at 22 weeks’ gestation [11]. Following delivery, women identified as high-risk can be assessed for ongoing cardiometabolic risk factors and offered lifestyle support [14,15].

**Figure 1:**
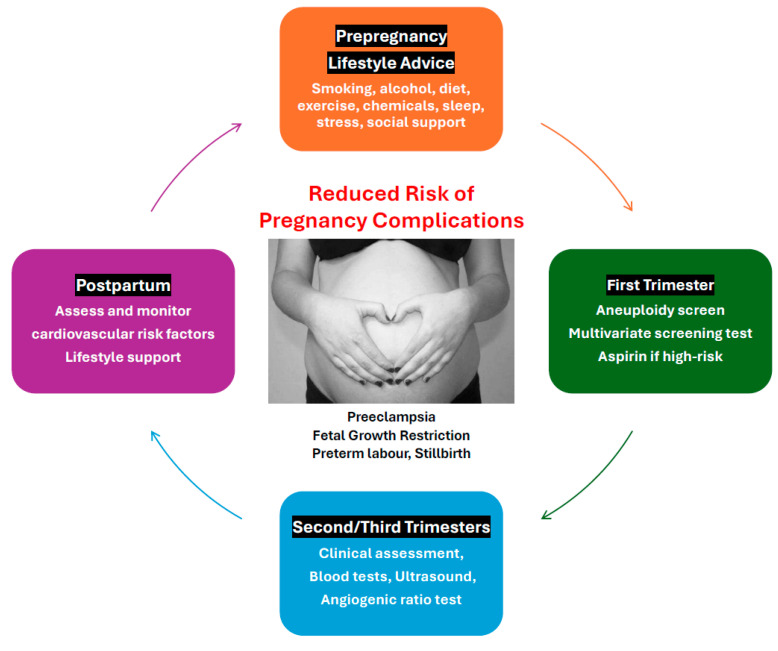
Components of the multimodal screening and medical management model.

This review outlines a multimodal approach that aims to reduce the risk of pregnancy complications, decrease social disruption, hospitalization, and time away from work and family, and reduce healthcare costs. The recommended strategy involves coordinated preconception, antenatal, and postpartum interventions (Figure 1).

## 2. Obstetric Syndromes

### 2.1. Pre-Eclampsia

Hypertensive disorders of pregnancy, including gestational hypertension and PE, result in significant maternal and fetal morbidity and mortality worldwide. Pre-eclampsia affects 2–7% of pregnancies globally and is responsible for 70,000 maternal deaths and 500,000 fetal/neonatal deaths every year [10]. Severe PE occurs in 1–2% of pregnancies and complications associated with this condition account for 15% of direct maternal deaths and 10% of perinatal deaths. Pre-eclampsia is the indication for 20% of labor inductions and 15% of Caesarean sections and accounts for 5–10% of preterm deliveries. Early-onset PE (<34 weeks) is less common than late-onset but accounts for significantly greater PE-related morbidity, mortality, and healthcare costs [16]. Women with a history of PE have a significantly increased risk of cardiovascular disease, metabolic disorders, chronic hypertension, renal disease, and dementia, later in life [14,17,18,19,20]. In addition, the experience of PE can be traumatic to women, their partners, and their support networks [21].

Many of the features of PE are non-specific (headache, visual disturbance, abdominal pain), and diagnoses based on clinical signs (hypertension and proteinuria) and symptoms are subjective and poor predictors of adverse outcomes [22]. Approximately 30% of all pregnancies will be evaluated for PE and require repeat hospital admissions and increased antenatal surveillance, which significantly add to healthcare costs [11]. Early identification of PE using predictive screening models and prophylactic treatment with lifestyle interventions and aspirin represents a significant advance in perinatal care [11,23].

#### 2.1.1. Screening for Pre-Eclampsia

Screening models for the detection of obstetric syndromes have primarily focused on identifying women at high risk of developing PE [24] and FGR [25]. First-trimester multivariate screening (maternal factors, mean arterial pressure [MAP], uterine artery pulsatility index [UtAPI], and pregnancy-associated plasma protein A or placental growth factor [PlGF]) are effective predictors of PE, having detection rates of 90% for early-onset PE (<34 weeks’ gestation), 75% for preterm PE (<37 weeks), and 42% for term PE, with a false-positive rate of 10% [10,25,26,27]. The effectiveness of the multivariate algorithm for detecting FGR is 50% [25,28].

Circulating angiogenic proteins, soluble fms-like tyrosine kinase-1 (sFlt-1), and PlGF have an important biological role in the pathophysiology of PE, are expressed prior to the onset of clinical signs and symptoms, and can be used in the prediction of early-onset PE [11,29]. The serum angiogenic factors can be assessed from 22 weeks’ gestation and have a high negative predictive value (NPV) to rule out PE in asymptomatic high-risk women and in women suspected of having PE [11]. The positive predictive value (PPV) to predict PE and/or adverse outcomes within 4 weeks is 65% when the cut-off of 38 is exceeded [11,29]. The angiogenic ratio test (sFlt-1/PlGF) can be used to exclude other conditions that mimic PE (e.g., non-HELLP thrombocytopenia, chronic hypertension, chronic kidney disease) [11,30,31], and can help discriminate between constitutional small-for-gestational-age (SGA) and growth-restricted fetuses [32]. Prediction of adverse outcomes is improved when angiogenic markers are combined with clinical, laboratory, and ultrasonographic data, to guide management [33].

#### 2.1.2. Screening for Preeclampsia Using Machine Learning (ML) Models

It is recognized that multivariate PE prediction models do not optimally predict all subtypes of PE, generally have a lower PPV than NPV, and will be more readily implemented in middle- and high-resource settings [34]. Greater than 99% of maternal deaths related to PE occur in low- and middle-income countries and the burden of adverse outcomes is spread across gestation [35]. In contrast, perinatal outcomes are largely related to gestational age at delivery. Machine learning (ML) models have the potential to be used globally for the prevention of complications related to obstetric syndromes.

Machine learning models are suitable for managing many variables and can be used for screening or diagnosis of PE. ML models can use combinations of maternal characteristics, medical history, antenatal serum biomarkers, laboratory data, ultrasound results, medications prescribed during pregnancy, and electronic health records, and can be used in real-world settings where available data are often incomplete [36,37]. A recent systematic review of 4 ML screening models found high predictive performance using routine early pregnancy information [37]. To date, there are no clinical trials that compare ML models to the currently recommended multivariate models.

A recent study by Li et al. used ML models to predict PE from clinical data obtained at the first antenatal visit [38]. The best predictive feature of the 38 clinical parameters assessed was fasting blood glucose, followed by mean blood pressure, and body mass index (BMI). These findings are consistent with the large body of research supporting the role of lifestyle and nutritional factors in the development of pregnancy complications (discussed in Section 2.1.5).

Machine learning models can also be used in women presenting with PE to rule out or rule in women at risk of adverse maternal outcomes in the following 48 h. Montgomery-Csoban et al. recently developed a novel ML-based time-of-disease model using routinely available data (health records, demographic and clinical data) [36]. The development dataset was derived from published data from low-, middle-, and high-income countries. The Pre-eclampsia Integrated Estimate of Risk-ML (PIERS-ML) model was externally validated in women hospitalized with PE and accurately identified women from low- to high-risk categories. The PIERS-ML model also identified women at very low risk of developing eclampsia and stillbirth. Some diagnostic ML models have included angiogenic markers in their algorithm [39]. Whether these models can improve the predictive performance of angiogenic markers used in conjunction with current management approaches, as recommended in our algorithm, has yet to be determined.

In the future, the further development of ML algorithms using large population-based datasets may improve the predictive performance of artificial intelligence-assisted models and thereby the prediction of specific subtypes of PE (such as late-onset) and other pregnancy syndromes associated with placental pathology.

#### 2.1.3. Placental Pathology in Pre-Eclampsia

The characteristic features of defective placentation in PE, such as incomplete remodeling of the junctional zone segment, atherosis of the decidual basal arterioles, and spiral artery thrombosis, are also found in other obstetric syndromes such as FGR, preterm labor with intact membranes, preterm pre-labor rupture of the membranes, placental abruption, preterm labor, and stillbirth [2,3,40,41,42,43,44]. These changes were initially identified in morphological studies, then more recently by functional investigations (doppler flow, uteroplacental perfusion, and biochemical and immunological studies), and electron microscopy [2]. Spiral artery remodeling commences in the first trimester of pregnancy when decidual natural killer cells and macrophages initiate disorganization and fragmentation of the vascular smooth muscle resulting in vessel dilatation [45,46]. This is followed by endovascular trophoblast invasion and more proximal vessel dilatation, in the second trimester, which extends into the myometrial segment of the spiral arteries and terminal radial arteries [46]. Failure of deep placentation during the second trimester is a common pathological feature in obstetric syndromes [2,3,47].

#### 2.1.4. Impact of Maternal Pathophysiology on Placentation in Pre-Eclampsia

Underlying maternal pathophysiological states such as chronic inflammation [48,49,50,51], insulin resistance [52,53,54], and hyperandrogenemia [55,56,57,58,59], alter placental metabolism and physiology and have a significant impact on placental development and function [4,7,60]. Laboratory cell culture, animal, molecular, human epidemiological, and interventional studies demonstrate clear associations and mechanistic links between maternal pathophysiology and placental dysfunction in PE and other obstetric syndromes [4,7,60].

Normal placental development is dependent on bidirectional feto-maternal communication signals such as cytokines, exosomes, extracellular vesicles, transcription factors, and hormones [7,61]. These signals are influenced by sperm and oocyte genetics, epigenetics, and metabolic factors [62,63,64], the physiological state of the maternal decidua, and the underlying maternal systemic metabolic and hormonal environment [7,65]. Disturbance of any of these components of normal physiology can lead to abnormal feto-maternal dialogue, deficient trophoblast invasion, altered spiral artery remodeling, and metabolic dysregulation that may all contribute to the common pathophysiological changes seen in obstetric syndromes [66,67,68,69].

#### 2.1.5. Lifestyle Factors in Pre-Eclampsia

Lifestyle factors have been extensively investigated for their role in modifying the risk of pregnancy complications [4,70]. An evidenced-based review by an international group of experts identified 78 maternal risk factors that were associated with the development of PE [71]. This comprehensive review of 2 umbrella reviews and 22 meta-analyses identified a number of lifestyle-related risk factors associated with PE. The study found that obesity (BMI > 30 kg/m^2^) was the strongest risk factor and had a “definite” association with PE based on high-quality evidence [71]. An evidence review of nutritional determinants of PE found that healthy maternal diet patterns (containing fruits, vegetables, whole-grain foods, fish, and chicken) were associated with a 22% reduction in the development of PE (Odds Ratio [OR]: 0.78, 95% confidence interval [CI] 0.70–0.86) [8,72]. Consumption of maternal dietary patterns high in ultra-processed foods and added sugars conferred a 28% increased risk of developing PE (OR: 1.28, 95% CI 1.15–1.42) [8,73].

There is significant overlap in the risk factors for PE and other obstetric syndromes [74]. Obesity is recognized as the most significant risk for PE and is associated with chronic inflammation, insulin resistance, and hyperandrogenemia, all of which can contribute to placental dysfunction [4,8]. International and national guidelines recommend lifestyle interventions, such as diet [8] and exercise [9] for the management of women with PE. The previous emphasis on smoking, alcohol, diet, and exercise has expanded to include stress, sleep, community engagement, social support, environmental chemicals, and the effects of climate [75,76,77,78,79,80]. Following a healthy lifestyle has been found to reduce the incidence of PE [72,81], preterm birth [72], gestational diabetes [72,81,82], gestational weight gain [83], SGA [84], and other pregnancy complications [85]. Nevertheless, lifestyle interventions are difficult to perform in pregnancy and not all studies have shown positive results [83,86]. The expanded list of lifestyle factors needs to be evaluated in large population-based intervention studies.

#### 2.1.6. Population-Attributable Risk of Pre-Eclampsia from Modifiable Risk Factors

Although some of the risk factors associated with PE are clinically important and identify women at significantly increased individual risk, they may only make a small contribution to the total burden of PE in the population [87]. One way to assess the broader impact of risk factors is to assess the population-attributable risk or proportional contribution of a risk factor to the entire population [88]. For example, it is important to identify women with a history of antiphospholipid syndrome for individual assessment and surveillance, but antiphospholipid syndrome was found to have one of the lowest population-attributable risk fractions of 0.18% with a 95% CI of 0.08 to 0.33%, for PE. The determination of the population-attributable risk can also provide important information that informs public health policy and prevention programs [71,88].

It is therefore important to identify women at increased individual risk, determine the population-attributable risk, and assess whether the relationship between the risk factor and PE is modifiable [71]. A large systematic review of cohort studies with more than 1000 participants evaluated the risk of PE in relation to common clinical risk factors in pooled data from 25 million women [87]. The investigators emphasized the importance of population-attributable risk and found that many common risk factors have a modifiable component. The pooled relative risk was used to calculate the population-attributable fraction for PE in relation to 16 common clinical risk factors. Nulliparity had the greatest population-attributable fraction (32.3%, 95% CI: 27.4–37%). When considered as a group, modifiable risk factors including pre-pregnancy BMI > 25 (23.8%, CI: 22 to 25.6%) and pregestational diabetes (3.7%, 95% CI: 3.1 to 4.3%) made up 27.5% of the population-attributable risk. It was noted that other risk factors that are linked to obesity, such as chronic hypertension, could also be reduced by a reduction in pre-pregnancy BMI, which would increase the proportion of modifiable risk for the development of PE [87]. In addition, other common lifestyle and metabolic-associated risk factors not assessed in this review, such as insulin resistance, polycystic ovary syndrome, and metabolic dysfunction-associated fatty liver disease, suggest that the modifiable population-attributable risk for PE would be significant.

**Figure 2:**
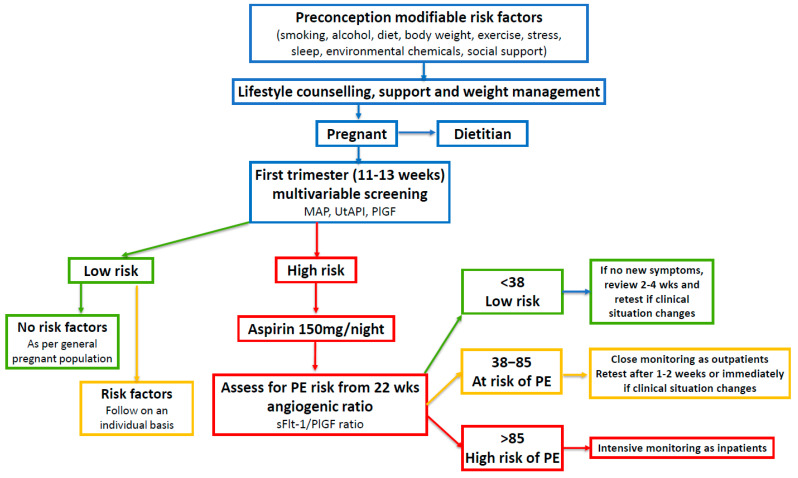
Risk assessment and management of women before and during pregnancy. First-trimester multivariable screening is based on references [10,24,26] (see https://fetalmedicine.org/research/assess/preeclampsia/first-trimester, accessed on 3 May 2024). The use of the aFlt-1:PIGF ratio is based on references [10,11,97,98,99]. Mean arterial pressure (MAP); uterine artery pulsatility index (UtAPI); placental growth factor (PlGF); soluble fms-like tyrosine kinase-1 (sFlt-1). Modified with permission from Parker et al. [4].

A subsequent hierarchical review of the relationship between 78 risk factors and PE by an expert advisory group also emphasized the importance of population-attributable fraction related to modifiable risk factors [71]. It is recognized that modifiable risk factors also contribute to fetal growth restriction [89,90], preterm labor [91,92], premature rupture of the membranes [93,94], and stillbirth [95,96]. The implementation of a multimodal intervention model that includes lifestyle advice, multivariate screening, aspirin prophylaxis, and assessment of the serum angiogenic ratio, therefore has the potential to detect and reduce morbidity and mortality related to many obstetric syndromes, as has been demonstrated with PE and FGR (Figure 2).

### 2.2. Fetal Growth Restriction (FGR)

Fetal growth restriction is influenced by maternal, fetal, and placental factors, and is a significant cause of perinatal morbidity and mortality [100,101]. Between 5 and 10% of pregnancies are complicated by FGR making it the second leading cause of perinatal mortality in babies without congenital anomalies. In addition, FGR is responsible for 30% of stillbirths [100]. Placental insufficiency is considered the main cause of FGR, and a variety of screening models related to placental function have been investigated [102,103,104]. Both first-trimester multivariate and second-trimester angiogenic assessments are predictive of early-onset FGR (<34 weeks), albeit at lower detection rates than those found in PE [25]. Early-onset FGR is a significant cause of iatrogenic preterm delivery and early detection of FGR is also important for reducing the incidence of stillbirth.

Many lifestyle interventions have been investigated for their effect on fetal growth and well-being. A systematic review found that unhealthy dietary patterns (high intakes of refined grains, processed meat, high saturated fat, or sugar) were associated with lower birth weight (mean difference: −40 g; 95% CI: −61 to −20 g) [105]. Physical activity during pregnancy has been shown to reduce the risk of gestational diabetes (by reducing blood sugar levels), decrease the risk of PE, lower the risk of Caesarean section, and reduce the severity of prenatal depression [81,82,106,107]. However, it is recognized that some modification of exercise routines may be necessary to accommodate maternal anatomical and physiological changes in pregnancy [108]. Low to moderate-intensity endurance and resistance training are associated with beneficial maternal and fetal effects [108]. The evidence suggests that high-intensity and volume weight-bearing and aerobic activity should be avoided, particularly during the third trimester, as it may contribute to lower birth weight [108,109,110]. Many national guidelines now contain specific advice regarding the frequency, duration, and intensity of exercise that is recommended in pregnancy [9,111]. These recommendations form part of a comprehensive multimodal intervention model.

### 2.3. Preterm Labor and Premature Rupture of the Membranes

Preterm birth, defined as delivery before 37 weeks’ gestation, occurs in 10.6% of pregnancies globally and is the leading cause of perinatal morbidity and mortality in the absence of congenital anomalies [112]. Preterm labor, with intact membranes or following premature rupture of the membranes, results in two-thirds of preterm births, and PE and FGR account for one-third [113]. Acute chorioamnionitis, as a cause or consequence, is the most common placental lesion in women with spontaneous preterm labor and vascular lesions are the second most common [3]. Preterm labor and premature rupture of the membranes are associated with defective placentation in common with other obstetric syndromes [2,3]. Since there is a significant overlap in the incidence of these conditions with other obstetric syndromes, interventions that reduce the impact of PE and FGR may also lower the incidence of preterm birth.

Maternal nutrition is a major determinant of birth outcomes and offspring health later in life [114]. A systematic review of observational studies that investigated the effect of dietary patterns in pregnancy found that healthy dietary patterns (high intakes of vegetables, fruits, whole grains, low-fat dairy, and lean protein foods) were significantly associated with a lower risk of preterm birth with an odds ratio (OR) of 0.79 (95% CI: 0.68 to 0.91) [105]. The investigators noted that the healthy diet patterns identified in the review were similar to current dietary recommendations in many countries (United Kingdom, United States, Canada, and China). These data support the recommendations of the current multimodal model that lifestyle and dietary advice should be aligned with national food and nutrition guidelines.

### 2.4. Stillbirth

It has been estimated that there are two million stillbirths in the world each year [115]. The majority of stillbirths (84%) occur in low-/middle-income countries, and the causes differ due to socioeconomic factors, both between and within countries [95,116,117,118]. These include genetic and environmental factors, maternal and fetal co-morbidities, and placental dysfunction. Of these, placental dysfunction, be it acute (abruptio placentae) or chronic (placental insufficiency), is the largest and most clearly defined risk factor. Forty percent of stillbirths occur intrapartum and are probably preventable [95]. Nineteen percent of stillbirths are associated with maternal risk factors (nulliparity, pre-existing hypertension, increased BMI) that are known to be associated with maternal and placental vascular dysfunction [96]. Over 50% of stillbirths are therefore preventable and multivariable prediction models and angiogenic factor assessments have the potential to improve early detection of fetal problems and facilitate preventative intervention in this group of women [5].

An umbrella review of 69 systematic reviews examining factors associated with stillbirth found that maternal age, BMI, and prior adverse pregnancy outcomes (stillbirth, preterm birth, small-for-gestational-age) were better predictors than ultrasound or biochemical markers [5]. Nevertheless, components of the multivariate model were found to be associated with an increased risk of stillbirth. Placental growth factor had a strong association with stillbirth with an OR of 49.2 (95% CI: 12.7 to 191) and second-trimester UtAPI had an OR of 8.3 (95% CI: 3.0–22.4) [5]. A prospective real-world study of 979 high-risk pregnant women found low PlGF levels (<100 pg/mL) were associated with an increased risk of preterm birth, early-onset PE, and stillbirth (OR 15.9, CI: 7.6–33.3). In addition, low PlGF levels were found to distinguish between placental and fetal causes of stillbirth [119].

Angiogenic factors have also been found to be of value in risk assessment for predicting stillbirth [119,120]. Chaiworapongsa et al. performed a prospective cohort study of 12 pregnant women and found that a reduced PlGF to soluble vascular endothelial growth factor receptor-1 (sVEGFR-1: also known as soluble fms-like tyrosine kinase-1) ratio at 34 weeks had a likelihood ratio of 14 for the prediction of subsequent stillbirth [120]. A cross-sectional study that included 44 women with unexplained fetal death, found a significantly higher concentration of plasma sVEGFR-1 (*p* = 0.04) than in normal pregnant women [121]. Future prospective studies will be required to investigate the predictive ability of combined multivariate first-trimester screening with second-trimester monitoring using angiogenic ratios to predict and reduce rates of both unexplained and syndrome-related stillbirth.

Population-level interventions, such as control of malaria and syphilis and optimizing nutrition, may play a significant role in stillbirth prevention at a global level [122]. A systematic review of behavioral and nutritional interventions before and during pregnancy concluded that many antepartum stillbirths are preventable through dietary and environmental interventions, and improved antenatal management of high-risk women [122]. A large cohort study from the United Kingdom found that potentially modifiable risk factors (maternal obesity, smoking in pregnancy, and FGR) were associated with over half of all stillbirths [123]. Therefore, the available evidence suggests that a combined multimodal approach that includes lifestyle and dietary advice also has the potential to reduce the incidence of stillbirth.

Additionally, there have been suggestions that aspirin may have a role in the prevention of stillbirth. Until recently, reported clinical trials have been underpowered to detect a reduction in this risk [13,95]. Now, a large multicenter stepped wedge cluster randomized controlled trial investigating the efficacy of the first-trimester screen-and-prevent strategy, has found that aspirin prophylaxis in high-risk women resulted in a 66% reduction of perinatal death (OR 0.34, 95% CI: 0.12 to 0.91) [124]. The identification of women at increased risk of pregnancy complications on first- and second-trimester screening facilitates increased surveillance and would be expected to help identify fetal compromise prior to stillbirth in some women. Taken together, the available evidence suggests that implementation of the multimodal model may reduce the incidence of stillbirth. This important area of perinatal research should be a priority in future large prospective trials.

## 3. Medical Management with Acetylsalicylic Acid (Aspirin)

Prophylactic low-dose aspirin therapy has been shown to be both efficacious and cost-effective for preventing pregnancy complications [16]. When aspirin is initiated in early pregnancy (<16 weeks’ gestation), it is associated with a significant reduction in early-onset PE [13], early-onset FGR [125], and preterm birth [16]. Women identified as high-risk on first-trimester multivariable screening can be offered prophylactic low-dose aspirin, which can be taken at night and continued until 37 weeks’ gestation. High-risk women can be followed with angiogenic factor assessment from 22 weeks’ gestation in conjunction with the current standard of care. Studies have shown that the reduction in the risk of complications is dependent on high rates of compliance with aspirin treatment [126]. Women with less than 90% adherence have a greater rate of PE (OR 2.3, 95% CI: 1.2–11.6, *p* = 0.03), FGR (OR 5.8, 95% CI: 1.2–8.3, *p* = 0.001), and preterm birth (OR 5.2, 95% CI: 1.5–8.7, *p* = 0.008) [127]. Effective education and compliance-aiding strategies are therefore of utmost importance in clinical practice.

Recent preliminary studies have investigated the possibility of ceasing aspirin therapy at 24 to 28 weeks’ gestation in high-risk women, if the angiogenic ratio and/or UtAPI are normal, to reduce side effects, and increase compliance and convenience [128,129]. A multicenter randomized trial (StopPRE) investigated whether aspirin (150 mg) could be discontinued at 24–28 weeks’ gestation if the angiogenic ratio was normal (<38) [128]. There was no significant difference in the incidence of pre-term PE in women who ceased aspirin at 24–28 weeks’ (1.48%, 7/473) compared with women who continued aspirin until 37 weeks’ gestation (1.73%, 8/463) (absolute difference −0.25%; 95% CI: −1.86% to 1.36%). The investigators found a higher incidence of minor antepartum hemorrhage in the group that continued aspirin until 37 weeks compared with those who discontinued treatment at 24–28 weeks’ gestation (12.31% vs 7.61%; absolute difference, −4.70; 95% CI: −8.53 to −0.87) [128]. A secondary analysis of the StopPRE trial showed that discontinuation of aspirin at 24–28 weeks in women with a UtAPI less than the 90th percentile was not inferior to continuing aspirin until 37 weeks’ gestation [129]. These data also suggest that there is a significant therapeutic effect of aspirin during the second trimester of pregnancy that corresponds to the period of deep placentation that is known to be a common pathological feature in obstetric syndromes [2]. Further intervention studies are required to investigate the reproducibility and generalizability of these results in diverse population groups [130].

## 4. Integrated Clinical Management to Reduce Pregnancy Complications

The proposed model is only one component of a comprehensive clinical management strategy to ensure high-quality pregnancy care and prevent complications. Lifestyle recommendations need to be easy to understand, succinct, and follow national and international guidelines [8,9]. Antenatal caregivers will need to be informed about the performance and interpretation of the new screening tests, and protocols for the measurement of MAP need to be implemented [131], ultrasonographers will be required to learn techniques for assessing UtAPI [132], serum biochemical tests need standardization and monitoring for compliance [133], and clinicians will be required to learn how to integrate components of the model into routine clinical practice. The angiogenic ratio test should be used in conjunction with ultrasound scans, usual blood tests, fetal monitoring, and clinical assessment (Table 1) [9].

### 4.1. Practical Aspects of Implementing First-Trimester Multivariate Screening

The Fetal Medicine Foundation (FMF) first-trimester multivariate risk assessment model is generally used worldwide for the prediction of PE and FGR [10,25,104,140]. This model has been extensively studied, has undergone successful internal and external validation, and is continually re-evaluated using real-world data [133,141]. Multivariate testing is superior to risk factor-based models for identifying high-risk women and allows individualized antenatal care [142,143]. Maternal risk factors and biomarkers can be entered into an online risk calculator free of charge at https://fetalmedicine.org/research/assess/preeclampsia, accessed on 3 May 2024.

The FMF screening test has been endorsed by the International Federation of Gynecology and Obstetrics [27], the International Society for the Study of Hypertension in Pregnancy [10], and many National Obstetric Societies [9]. In order to maintain optimal screening performance, it is important to follow standardized methods for performing the required biophysical (MAP, UtAPI) and biochemical (PlGF) measurements.

#### 4.1.1. Measurement of Mean Arterial Blood Pressure (MAP)

Determining MAP antenatally is inexpensive, non-invasive, quick, and can be performed with minimal training. However, its effectiveness depends on various factors such as the population studied, user training, measurement accuracy, and the protocols for intervention based on the results [133]. Inaccurate measurements of MAP affect the performance of the screening test and impact the risk estimate given to the patient [131]. Standardized measurement protocols have therefore been developed to limit data errors entered into the FMF risk calculator.

Mean arterial blood pressure is measured with women sitting with their backs against the seat, legs uncrossed, and arms supported at the level of the heart. The correct cuff size is selected, and blood pressure is measured in both arms simultaneously using a validated automated device. Two readings are taken from each arm, 1 min apart, and MAP is calculated from the average of the 4 measurements [131]. Automated blood pressure devices require calibration at regular intervals to ensure reliable measurements over time [144].

#### 4.1.2. Measurement of Uterine Artery Pulsatility Index (UtAPI)

The reproducibility and reliability of UtAPI assessment are dependent on the use of standardized protocols that take measurements at defined anatomical locations using specific ultrasound machine settings [132]. A transabdominal ultrasound transducer is used to obtain a sagittal section of the uterus at the level of the internal cervical os. The uterine arteries are identified using Color Doppler flow mapping followed by pulsed wave Doppler measurement of UtAPI and peak systolic velocity when 3 consecutive waveforms are obtained. Standardized transducer positions and Doppler settings are employed [133,145].

Ultrasonographers require specific training in the UtAPI measurement technique followed by regular assessment of their results for continued accreditation [132]. This procedure has been shown to reduce operator- and technique-dependent measurement variability, and improve detection rates for PE [146,147]. Measurement of UtAPI can be taken between 11 and 14 weeks’ gestation at the same time as the first-trimester scan.

#### 4.1.3. Measurement of Placental Growth Factor (PlGF) and Compliance Monitoring

Standardized protocols are required to minimize variations in the measurement of PlGF that can arise due to changes in reagent batches, fluctuations in temperature, deviations from manufacturer protocols, and the absence of a continuous quality control process [133,148,149]. Automated assays allow standardized measurements with rapid availability of results [148]. Measurement of PlGF can be conducted on the same blood sample as routine blood tests for first-trimester aneuploidy screening.

A comparison of 3 commercially available automated immunoassays found that there was a considerable difference between raw data values between different platforms, which was likely to be clinically significant [148]. The authors recommended that reference ranges specific to each platform should be reported with raw data values when PlGF measurement is used in clinical practice. Conversion of raw data to multiples of median values allows direct comparison of results between different platforms. Analyzers need frequent calibration and results are regularly monitored to ensure consistency.

#### 4.1.4. Integrating Angiogenic Ratio Testing into Clinical Practice

There is now international consensus that all pregnant women should be offered first-trimester multivariate screening [10,27]. Approximately 10% of women will be classified as high-risk based on maternal factors, placental biomarkers, MAP, and UtAPI. The data are entered into the FMF risk calculator and high-risk women can be offered prophylactic treatment with low-dose aspirin [13]. Asymptomatic high-risk women may then be followed with monthly sFlt-1/PlGF ratio tests from 22 weeks (Figure 2) [11].

An angiogenic ratio <38 can rule out the onset of PE for 1 week with an NPV of 99.3% and up to 4 weeks with an NPV of 94.3% [11]. This can provide reassurance to clinicians and women for continued outpatient management [150]. Women with an intermediate ratio result of 38–85 require increased outpatient monitoring. This may include clinical assessment, ultrasound, cardiotocography, blood tests, repeat blood pressure measurement, and a repeat angiogenic ratio test in 1–2 weeks, or sooner, if the clinical situation changes. Women with a sFlt-1/PlGF ratio >85 require intensive monitoring, usually as inpatients.

Most of the research on angiogenic factors has so far been related to predicting, diagnosing, and/or managing PE and its complications, as well as assessing the severity and the associated rate of clinical deterioration in patients with PE. However, emerging evidence suggests that altered levels of the sFlt-1/PlGF ratio or PlGF itself are also associated with FGR, preterm birth, and stillbirth [151,152,153]. This reflects the common pathogenesis of such outcomes as often being related to placental dysfunction, of which angiogenic biomarker imbalance is a feature.

Additionally, angiogenic factor measurement may be of clinical value in the differential diagnosis of PE-like conditions that may occur during pregnancy [11,30,31,97]. These include presentations involving exacerbations of chronic hypertension, systemic lupus erythematosus, diabetic nephropathy, renal transplant rejection, and other chronic kidney diseases, as well as new presentations of conditions manifesting as hypertension (e.g., phaeochromocytoma), liver dysfunction (e.g., hepatitis), proteinuria (e.g., nephrotic syndrome) or thrombocytopenia (e.g., idiopathic thrombocytopenic purpura).

At a research level, these angiogenic biomarkers will also be valuable in helping the selection of suitable trial entrants for studies examining the treatment and/or management of PE and related disorders. For example, an sFlt-1/PlGF ratio above a certain cut-off level could be an inclusion criterion for recruitment to a study, thereby optimizing the number of suitable at-risk patients enrolled and facilitating sufficient sample size achievement in a more cost-effective manner than might otherwise be the case.

Also, it is worthy of note that support for the use of angiogenic biomarkers by national authorities has been gathering momentum [154,155], and health economic evaluations of their use in practice have been consistently positive [156]. A review of nine studies that investigated the cost-effectiveness of the use of diagnostic angiogenic biomarkers in women suspected of having PE found that all studies demonstrated cost savings [156].

Overall, the expanded use of angiogenic factor measurement as a standard part of antenatal care has the potential to improve maternal and perinatal outcomes in pregnancies complicated by placental dysfunction. An elevated angiogenic ratio facilitates early diagnosis of placental dysfunction and effective intervention can mean a cost-beneficial use of limited health funds. Equally important, having a normal angiogenic ratio excludes a diagnosis of placental dysfunction and can mean avoiding ineffective overuse of scarce health resources.

## 5. Strengths and Limitations of the Current Review

### 5.1. Strengths

Individual components of the proposed model have been extensively evaluated in case-control, prospective, randomized, and real-world implementation studies. There is a large body of research investigating the role of nutrition in promoting healthy pregnancy and preventing complications. In summary, systematic reviews of observational studies, expert reviews, and national and international guidelines, support dietary and exercise recommendations for lifestyle-based interventions before, during, and after pregnancy. Multivariate and angiogenic factor screening strategies have been extensively evaluated over two decades in a variety of ethnic populations and clinical environments. Over 75 randomized trials have consistently shown a significant reduction in pregnancy complications, such as PE and fetal growth restriction, using prophylactic low-dose aspirin. The proposed multimodal model is the first time all of the individual components have been combined into a sequential algorithm that can be integrated into existing clinical practice structures.

### 5.2. Limitations

The implementation of this model has some limitations. Education and training of healthcare practitioners in various aspects of the proposed model will be required. Protocols for the measurement of mean arterial blood pressure need to be implemented. Ultrasonographers will need to be upskilled and accredited in techniques for assessing uterine artery pulsatility index, which is a measurement taken at the time of the first-trimester ultrasound. Protocols to ensure that laboratories comply with quality control of serum biomarker assays will also be needed. Doctors managing pregnant women will require information about how to integrate angiogenic ratio results into existing clinical management practices. The multivariable model has a 10% false positive rate, so some low-risk women will be assessed as high-risk, and adequate counseling will be required. Equity of access to qualified professionals will take time during the training and implementation phase and real-world evaluation of the combined multimodal model will be essential.

## 6. Conclusions

Pregnancy conditions resulting from placental dysfunction may complicate up to 30 million pregnancies worldwide annually. Implementation of a multimodal integrated management strategy using lifestyle, screening, and medical treatment has the potential to significantly reduce pregnancy complications, decrease maternal and perinatal morbidity and mortality, limit transgenerational transmission of chronic disease, reduce future maternal cardiometabolic risk, decrease healthcare-related costs, and improve quality of life. Translation of validated components of this model into clinical practice should be a global healthcare priority.

## Figures and Tables

**Table 1:** Characteristics of the Multimodal Model.

Intervention	Recommendation	Intervention	References
Nutrition advice	As per national dietary guidelines	PRECISE, Australia, USA, Canada, UK, China	[8,9,134,135,136,137]
Exercise advice	According to expert advisory groups	Endurance, strength, stretching	[9,138]
Multivariate screening	First-trimester assessment as per Fetal Medicine Foundation algorithm	Maternal factors, MAP, UtAPI, PlGF at 11–14 weeks’ gestation	[24,26]
Aspirin	As per national guidelines	81–150 mg at night	[13,139]
Angiogenic ratio screening	Serum sFlt-1/PlGF ratio as per protocol	Measurement from 22 weeks’ gestation	[11,97,99]
Clinical management	Multidisciplinary team	Lifestyle advice, clinical assessment, blood tests, blood pressure, CTG, ultrasound, assess postpartum	[9,14]

Abbreviations: PRECISE = Pregnancy care integrating translational science everywhere; USA = United States of America; UK = United Kingdom; MAP = mean arterial pressure; UtAPI = Uterine artery pulsatility index; PlGF = Placental growth factor; sFlt-1 = soluble fms-like tyrosine kinase-1; CTG = cardiotocography.

## Data Availability

Not applicable.

## Abbreviations

BMI	body mass index
CI	confidence interval
CTG	cardiotocography
FGR	fetal growth restriction
FMF	Fetal Medicine Foundation
HELLP	hemolysis, elevated liver enzymes, and low platelets
KG	kilogram
MAP	mean arterial pressure
M^2^	meter squared
ML	machine learning
NPV	negative predictive value
OR	odds ratio
PE	preeclampsia
PIERS	pre-eclampsia integrated estimate of risk
PlGF	placental growth factor
PPV	positive predictive value
sFlt-1	soluble fms-like tyrosine kinase
SGA	small for gestational age
sVEGFR-1	soluble vascular endothelial growth factor receptor-1
UtAPI	uterine artery pulsatility index
